# Diagnostic journey and multimodal management of a rare urogenital rhabdomyosarcoma with rectovaginal fistula in an adolescent: a case report

**DOI:** 10.1097/MS9.0000000000001423

**Published:** 2023-11-07

**Authors:** Shailendra Katwal, Aastha Ghimire, Kriti Shrestha, Suban Amatya

**Affiliations:** a Radiologist, Department of Radiology, Dadeldhura Subregional Hospital, Dadeldhura; b Patan Academy of Health Sciences, Lalitpur, Nepal

**Keywords:** case report, multidisciplinary treatment, paediatric tumour, rhabdomyosarcoma, urogenital

## Abstract

**Introduction and importance:**

Rhabdomyosarcoma is a malignant tumour that originates from immature muscle cells and belongs to the category of soft-tissue sarcomas. It is predominantly diagnosed in children under the age of 6. This condition can manifest within the genitourinary tract and may exhibit non-specific symptoms such as changes in bowel habits and fever. Early detection and a comprehensive, multidisciplinary approach are essential to achieving more favourable outcomes. This report highlights an uncommon case of urogenital rhabdomyosarcoma in a 15-year-old girl, in addition to the presence of a rectovaginal fistula.

**Case presentation:**

A 15-year-old girl with presented with fever, altered bowel habits, and a lump in her lower abdomen, abdominal discomfort, and incomplete bowel evacuation. She also had faecal discharge from the vagina. Diagnostic imaging and biopsy confirmed urogenital rhabdomyosarcoma with a rectovaginal fistula. The patient is currently undergoing induction chemotherapy and is scheduled for radiation therapy and surgery.

**Clinical discussion:**

Rhabdomyosarcoma is a rare paediatric oncologic concern due to its aggressive nature and potential metastasis. The presentation varies based on age, tumour location, and metastasis presence. This patient presented with altered bowel habits, a pelvic mass and unusual feculent discharge, suggesting a rectovaginal fistula. Diagnostic imaging confirmed the diagnosis, and induction chemotherapy led to a positive response and reduced tumour size.

**Conclusion:**

Urogenital rhabdomyosarcoma is an aggressive malignancy with non-specific symptoms, making early diagnosis challenging. An accurate diagnosis requires high suspicion, imaging, and a biopsy. Multidisciplinary management, including surgery, chemotherapy, and radiation therapy, improves outcomes and improves paediatric patients’ prognosis and quality of life

## Introduction

Highlights

- A rare case of urogenital rhabdomyosarcoma in a 15-year-old girl, presenting with gastrointestinal and gynaecological symptoms.
- The study underscores the importance of early recognition and comprehensive multidisciplinary management to achieve successful treatment outcomes.
- Neoadjuvant chemotherapy and temporary faecal diversion played crucial roles in tumour size reduction and positive treatment response.

Rhabdomyosarcoma is a highly malignant tumour that arises from skeletal muscle cells (myoblasts), immature cells, and myogenic satellite cells and it is one of the most common soft-tissue sarcomas in children and adolescents, with a worldwide incidence of 4.5 cases per million among those younger than 20 years of age^1^. Besides that, it can also arise from other locations than skeletal muscle, like in our case, it was urogenital^1^. Almost 2/3 of cases are diagnosed in younger children, younger than 6 years of age. The male-to-female ratio is 1.4:1^2^. Genitourinary tract rhabdomyosarcoma occurs most commonly in the bladder, prostate, vagina, and uterus, and para testicular disease. Here, we present a rare case of urogenital rhabdomyosarcoma in a 15-year-old girl who presented with altered bowel habits and fever^3^. This study aims to highlight the unique presentation in this case and showcase the importance of early diagnosis and treatment of this aggressive malignancy.

## Case details

A 15-year-old girl presented to our outpatient department along with her mother with complaints of fever for 1 month, and altered bowel habits and a lump over the lower abdomen for 2 months. She was in her usual state of health 2 months ago when she started experiencing alterations in her usual bowel habits. The frequency and consistency of stool had altered. The symptoms progressively increased, and she was having seven to eight episodes of loose stool at the time of presentation, which was mixed with mucus and bright red blood. These episodes were accompanied by lower abdominal discomfort, which improved after defecation, and a sensation of incomplete bowel evacuation. However, she did not experience any changes in stool calibre, mass per anum, or early morning diarrhoea. A few days after the onset of these symptoms, her mother noticed an abdominal lump. According to her mother, she palpated the lump over the lower abdomen while trying to relieve the discomfort. The lump was approximately the size of her hand, non-painful to touch, and had not increased in size over the past two months. The patient also experienced intermittent fever for the past month. The maximum recorded temperature at home was 102° Fahrenheit, accompanied by sweating and an evening rise in temperature. However, she did not complain of chills and rigors. In addition to these symptoms, she reported the onset of a new symptom: passage of foul-smelling, blood-mixed, feculent discharge per vaginum over the last 2 weeks. Family members noticed a significant loss of appetite and weight, with a recorded weight reduction of five kilograms during this period. She also experienced easy fatigability, evident by her inability to perform daily activities without resting in between, and had a generalized feeling of weakness. There were no lumps noticed in other parts of her body, and she had no history of prior recurrent diarrhoea, arthralgia, or perianal diseases. The patient did not report abdominal pain, vomiting, haematuria, increased frequency of micturition, burning micturition, passage of faecal material during urination, back pain, or bone pain. Additionally, she did not complain of palpitations, loss of consciousness, altered sensorium, or abnormal body movements. She and her family had no history of tuberculosis, and she had never experienced symptoms such as cough, chest pain, shortness of breath, or hemoptysis. She had not encountered similar episodes in the past and had not undergone any surgical procedures. The patient was unmarried, not sexually active, a non-smoker, did not consume alcohol, and followed a balanced non-vegetarian diet. In her family history, her grandmother’s death due to an abdominal malignancy was mentioned, although the documentation was unavailable. Her parents and four siblings were in their usual state of health. She had reached menarche 5 months ago and had regular menstrual cycles without excessive bleeding or pain. Before visiting our centre, the patient had visited another hospital where an ultrasound-guided aspiration of serous fluid had been done and she had received intravenous antibiotics. After getting discharged on request from there the patient presented to our centre.

On examination, the patient was alert, oriented to time, place, and person, and appeared thinly built. Pallor was appreciated on palpebral conjunctiva and she had no signs of icterus, dehydration, or clubbing, and no lymph nodes were palpable. Her BMI was recorded to be 16.6 kg/m^2^. She was afebrile at presentation and had a blood pressure of 100/70 mm of Hg, pulse of 70 beats per minute, and regular, respiratory rate of 16 per min and she maintained a saturation of 95% in room air. A visible lump was observed in the hypogastrium and the right iliac fossa, with normal overlying skin and no visible pulsations. Upon palpation, a globular, firm, non-tender mass measuring 10×8 cm in size was noted. The mass extended from the hypogastrium and right iliac fossa to the pelvis, with regular upper and lateral borders. It was fixed and showed no movement in any planes. The prominence of the mass increased when the patient was in the knee-elbow position and decreased during the straight leg-raising test. No pulsations were felt, and the mass produced a dull sound on percussion. The rectal examination showed normal anal tone, an empty collapsed rectum, and a polypoidal palpable lesion at the tip of the finger. The upper margin of the growth was not palpable, and the mass did not bleed upon touch. An anterior firm to boggy, regular, non-tender swelling was felt from the 9 to 3 o’clock position, with normal overlying rectal mucosa. Upon applying pressure over the swelling, there was a passage of feculent discharge per vaginum, and the examining finger was stained with stool mixed with altered blood. Subsequently, proctoscopy revealed the lower margin of a non-friable polypoidal growth, visible approximately 7cm from the anal verge at the 1 o’clock position. A per vaginal examination was not performed. The rest of the examination was within normal limits.

**Figure 1:**
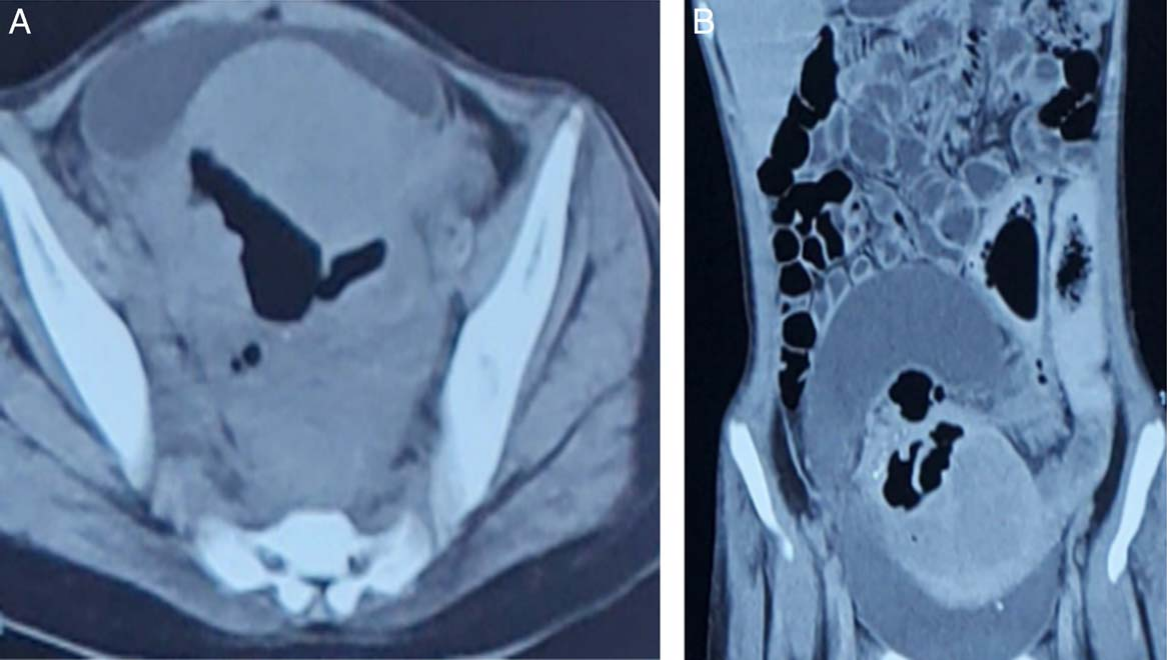
(A) Axial contrast-enhanced computed tomography (CT) image of pelvis showing homogenously enhancing soft-tissue density mass behind the bladder with indistinct fat plane to rectum. There is oblong air density extending from rectum to the mass. (B) Coronal contrast-enhanced CT image showing the thickened colon adjacent to the mass with indistinct fat plane. The heterogenously enhancing mass shows air content with surrounding hypodensity.

Based on the patient’s history and examination, a provisional diagnosis of rectal lymphoma with a rectovaginal fistula was made. To further investigate the condition, a complete blood count, renal function test, urine routine examination, chest X-ray, and ultrasonography of the abdomen were ordered [Table 1]. The complete blood count revealed a decreased haemoglobin level, and the ultrasonography showed a heteroechoic area measuring approximately 11.9×8×8.9 cm (459 cm^3^) in the pelvic cavity. Additionally, an adjacent hypoechoic area was observed extending from the pelvis up to below the umbilicus, with more prominence on the right side. This was followed by contrast-enhanced computerized tomography of the abdomen which showed an irregularly defined, multiloculated, thick, peripherally enhancing, walled heterogeneous collection measuring ~12.9×7.6×6.4 cm with multiple hypodense areas of air attenuation in non-dependant part within it as well as areas of fluid attenuation in dependant part and few foci of calcifications in the Pouch of Douglas. An associated long segment asymmetrical bowel was thickening involving the rectum measuring ~15 mm and circumferential thickening of sigmoid colon measuring ~8 mm was noted. Uterus appeared enlarged with associated altered attenuation representing secondary changes [Figure 1A, B].

**Table 1:** Haematological and biochemical findings of the patient

Examination	Result	Reference range
CBC—on day of presentation		
Total leucocyte counts	5.7 thou/ul	4.5–11.0×10^9^/l
Neutrophil	75	40–70%
Lymphocyte	20	20–45%
Monocyte	3	2–10%
Eosinophil	2	1–6%
Haemoglobin	8.4 g/dl	12–14 g/dl
Platelets count	203 000 thou/ul	150 000–450 000
RFT—on day of presentation		
Serum urea	18 mg/dl	6–24 mg/dl
Creatinine	0.46 mg/dl	0.4–1.4 mg/dl
Sodium	134 mmol/l	135–145 mmol/l
Potassium	3.8 mmol/l	3.5–5.5 mmol/l
Urine routine examination—on day of presentation		
Colour	Yellow	
Transparency	Clear	
Reaction	Acidic	
Albumin	1+	
Sugar	Nil	
Pus cells	0–2/HPF	
RBC	1–2/HPF	
Epithelial cells	3–4/HPF	

CBC, complete blood count; HPF, High Power Field; RFT, Renal Function test.

These findings were followed by a core needle biopsy of the mass, which confirmed rhabdomyosarcoma. Consequently, the diagnosis was changed to urogenital rhabdomyosarcoma with a rectovaginal fistula. The parents and the patient were informed about the diagnosis and counselled on the requirement of a multidisciplinary approach to treat this locally advanced malignancy. Induction chemotherapy was discussed and initiated, and the patient is currently undergoing treatment with Vincristine, cyclophosphamide and Actinomycin D, showing a positive response with a decrease in the size of the tumour. Vincristine was given at a dose of 1.5 mg/m^2^ at day 1, 8 and 15, cyclophosphamide was administered at a dose of 1.2 g/m^2^ on day 1 and Actinomycin D was given at a dose of 0.045 mg/kg on day 1. This regimen is planned to be repeated every 3 weeks for 44 weeks. To prevent the passage of faecal contents per vaginum, a temporary faecal diversion has been performed. The patient is scheduled for radiation therapy and excisional and reconstructive surgery after completing the induction therapy. Regular hospital visits for chemotherapy are being made, and the patient is participating in psychological support sessions to facilitate the process. However, the patient and the family are facing financial and psychological difficulties. The hospital administration has been providing the necessary assistance available, and they remain hopeful for a successful recovery.

## Discussion

Rhabdomyosarcoma (RMS) is the third most prevalent solid, extracranial tumour in children, following neuroblastoma and Wilms tumour^4^. It accounts for ~4% of paediatric cancer cases^5^. RMS originates from embryonal mesenchyme, which has the potential to differentiate into skeletal muscle^4^. Although it can develop anywhere in the body, it most commonly affects the head and neck (26%) and genitourinary tract (22%) in children^4^. With the genitourinary RMS, there are two main groups based on the prognosis and treatment approach: bladder and prostate RMS and other sites, including para testicular, vaginal, vulva, perineum, and uterus. Those involving the bladder and prostate have a worse prognosis^6^.

The clinical presentation of rhabdomyosarcoma depends upon the patient’s age, site of lesion, and presence of metastasis^6^. Most common symptoms are due to the compressive effect of the tumour or associated lymph nodes^6^. Urinary obstruction, constipation, urethral strangury, and the presence of pelvic mass are common symptoms of urogenital rhabdomyosarcoma. Other non-specific symptoms include weight loss, fatigue, and low blood counts^6^.

Fistulas are abnormal or surgically made passages between a hollow body organ and the body surface, or between two hollow or tubular organs^7^. Rectovaginal fistulas are abnormal epithelial-lined connections between the rectum and vagina^7^. These fistulas can be acquired or as a cause of congenital malformation^7^. The common acquired cause can be due to necrosis of the rectovaginal septum or obstetric injury during childbirth. Infections such as anorectal abscess, Bartholin gland infection, as well as inflammatory bowel disease, can be possible causes of rectovaginal fistula^7^. Malignancies can also be another possible culprit. Rectal, uterine, vaginal, and cervical malignancies, with significant local extension or after being treated with radiation therapy can lead to the formation of rectovaginal fistulas^8^. The clinical presentation usually depends upon the size and location of the fistula^7^. The most frequent symptoms are the passage of feculent discharge per vaginum along with recurrent vaginitis and malodorous vaginal discharge^7^.

Our patient presented with similar findings of altered bowel movement and abdominal lump. Along with it, non-specific symptoms like weight loss, anorexia, and easy fatigability. She had a firm, non-tender lump over the hypogastrium, extending up to the pelvis. These symptoms are commonly seen in cases of urogenital rhabdomyosarcoma. She also had the presence of foul-smelling feculent discharge per vaginum. On Digital Rectal Examination, there was a polypoidal growth with the firm to boggy swelling palpable anteriorly, which on being compressed led to feculent discharge from the vagina. Although rectovaginal fistula can occur due to malignancies, it rarely occurs without any radiation or extensive local spread. It takes around 6 months to 2 years post-radiation for rectal ulcers to progress to fistula formation. Both rectovaginal fistula and rhabdomyosarcoma were simultaneously diagnosed in our patient.

Besides clinical examination, imaging also plays an important role in the diagnosis of both rhabdomyosarcoma and rectovaginal fistula. Ultrasound is usually the first line of investigation for any abdominal or pelvic mass. In ultrasound, rhabdomyosarcoma has non-specific features with well-defined, slightly hypoechoic, inhomogeneous mass, rarely with calcification^9^. In our case, the patient’s USG showed a hyperechoic area of 11.9×8×8.9 cm in the pelvic cavity, with an adjacent hypoechoic area extending from the pelvis up to below the umbilicus, causing moderate hydronephrosis over the right side. Contrast-enhanced computed tomography (CT) of the abdomen and pelvis is done due to the high incidence of retroperitoneal lymph node involvement^10^. The patient had an irregularly defined, multiloculated, thick peripherally enhancing walled, heterogeneous collection measuring ~12.9×7.6×6.4 cm with multiple hypodense areas of air attenuation in the non-dependent part and fluid attenuation in the dependent part. There were also a few foci of calcification in the pelvic cavity, predominantly in the pouch of Douglas. The lesion caused a mass effect leading to increased attenuation of the pelvic mesentery, engorgement of pelvic vessels, and shift of the sigmoid colon and descending colon. The mass also caused compression of the right ureter leading to right-sided moderate hydronephrosis. MRIs are also done showing non-specific signs with intermediate signals on T1-weighted images and intermediate to high signals on T2-weighted images, with strong enhancement^6^. Chest CT and bone should also be done to scan for possible metastasis^11^. Other new techniques, such as FDG/ PET scan in childhood rhabdomyosarcoma have a sensitivity of 77–100% for primary tumour, 62–77% for regional or distant disease, and a specificity of 83–95%. A study suggest whole-body MRI especially in the paediatric population for staging regarding concerns for ionizing radiation and ability to detect metastases as well as PET-CT since unusual metastatic lesions can be seen in RMS even in the absence of metastases to the more frequent sites^12^.

Supplementary diagnostic studies may be required for confirming the diagnosis of rectovaginal fistula but such confirmatory diagnostic studies are only done when the rectovaginal fistula eludes identification on physical examination or if the extent of disease is unknown^13^. Vaginal tampons can be visualized after the instillation of methylene blue enema^7^. The tampons are visualized after 15–20 of retaining the enema, and if there is no staining, the diagnosis of the rectovaginal fistula is doubtful^7^. Vaginography or CT with rectal contrast may be required for a better diagnosis of proximal fistulas^7^.

Factors like age at diagnosis, size of the tumour, resectability, metastasis, response to chemotherapy, etc determine the prognosis of this condition^14^. The standard treatment modalities include surgery, chemotherapy, and radiation therapy. Rhabdomyosarcoma can form in many different places in the body and the surgery will be different for each site. Radiation therapy is a cancer treatment that uses high-energy X-rays or other types of radiation to kill cancer cells or stop them from growing^14^. The type and amount of radiation therapy and when it is given depends on the age of the child, the type of rhabdomyosarcoma, where in the body the tumour started, how much tumour remained after surgery, and whether there is a tumour in the nearby lymph nodes^14^. External radiation therapy is usually used to treat childhood rhabdomyosarcoma but in certain cases, internal radiation therapy is used. Chemotherapy may also be given to shrink the tumour before surgery to save as much healthy tissue as possible^14^. This is neoadjuvant chemotherapy. Every child treated for rhabdomyosarcoma should receive systemic chemotherapy to decrease the chances for the cancer to recur^14^. The type of anticancer drug, dose, and number of treatments given depends on the age of the child and whether the child has low-risk, intermediate-risk, or high-risk rhabdomyosarcoma^14^. There are newer modalities of treatment undergoing trials which includes immunotherapy and targeted therapies^14^. The overall treatment circles round the multidisciplinary approach according to the areas affected. In our case the team for her treatment consisted of oncology, paediatrics, urosurgery, gynaecology and psychiatry. Their roles were in deciding and providing appropriate chemotherapy, tackling the adverse effects of the chemotherapeutic agents, ensuring nutrition, deciding and performing the surgeries and supporting the family and the patient through the emotional and psychological aspects of the diagnosis and treatment. Since the treatment of conditions like these involves multiple interventions and a long duration, the financial burden upon the family is overwhelming, especially in patient living in rural area of countries likes Nepal. Her treatment is currently being aided by charity and the social services at the hospital and the parents are still raising funds for her treatment.

## Conclusion

This case highlights the importance of considering urogenital rhabdomyosarcoma in adolescents presenting with altered bowel habits and lower abdominal lumps. Prompt diagnosis and a multidisciplinary approach are crucial for effective treatment. Aggressive nature of the tumour and possible metastasis makes early diagnosis very important. It mostly affects children making it more challenging for early identification and treatment. Along with the multidisciplinary approach to the treatment, addressing psychological aspects of the patient and the family and financial burden of disease is equally important for the successful recovery.

## Ethical approval

Ethical approval is not required for case reports in my institution (Patan Academy of Health Sciences, Bagmati Lalitpur) so ethical approval was exempted.

## Consent

Written informed consent was obtained from the patient for the publication of this case report and accompanying images. A copy of written consent is available for review by the Editor in chief of this journal on request

## Source of funding

None.

## Author contribution

S.K.: conceptualization, mentor and reviewer for this case report and for data interpretation. A.G.: contributed in performing literature review, writing the paper and editing. K.S.: contributed in writing the paper. S.A.: contributed in writing the paper. All authors have read and approved the manuscript

## Conflicts of interest disclosure

All the authors declare that they have no competing interest.

## Research registration unique identifying number (UIN)

Not applicable.

## Guarantor

Shailendra Katwal.

## Data availability statement

Not applicable.

## Provenence and peer review

Non commissioned, externally peer-reviewed.
